# Editorial: Recent Advances and Challenges on Big Data Analysis in Neuroimaging

**DOI:** 10.3389/fnins.2016.00505

**Published:** 2016-11-15

**Authors:** Jian Kang, Brian Caffo, Han Liu

**Affiliations:** ^1^Department of Biostatistics, University of MichiganAnn Arbor, MI, USA; ^2^Department of Biostatistics, Johns Hopkins UniversityBaltimore, MD, USA; ^3^Department of Operations Research and Financial Engineering, Princeton UniversityNew Jersey, NJ, USA

**Keywords:** neuroimaging, big data analytics

“…the most powerful computer in the world isn't nearly as intuitive as the one we're born with. So there is this enormous mystery waiting to be unlocked.”

—President Obama Announcing the BRAIN Initiative

In its Big Data to Knowledge initiative, the US National Institutes of Health notes the wealth of biomedical and behavioral information will greatly advance our understanding of human health, disease and treatment–only if new analytic tools are developed and the understanding of these new tools is broadly disseminated (https://datascience.nih.gov/bd2k). Big Data encompasses the study of data formats from long, in the sense of multitudes of subjects, and wide, in the sense of complex measurements across relatively few subjects. Brain imaging tends to be of latter category. However, it is essential for our field to prepare for the inevitability of both long and wide neuroimaging data.

The stakes couldn't be higher, as the promise of Big Data in neuroscience seems limitless. Recent advances in neuroimaging technology offers great hope for significant progress in furthering the understanding the human brain, with the potential to facilitate research in medicine, neuroscience, psychology, and many other disciplines. This technology enables the creation of massive amounts of high-resolution images, which capture the structure, function and composition of human brains. Parallels to brain imaging are often made with the scope, scale, scientific goals and importance of mapping and analyzing the human genome, and other “biomes” (proteome, transcriptome, microbiome). In fact, intra-brain structural and functional connections have their own portmanteau, the so called “connectome” (genome and connection). The implication of myriad of these new disciplines, including brain imaging, is the central idea of the measurement of the intrinsic, unique, fundamental, and personal measurements that will make true precision medicine a reality.

However, such breakthroughs in the development of effective personalized treatments of neurological and psychiatric disease require a massive effort in the: Measurement, informatics and analytic capacity to handle the large databases of subjects, increasingly fine temporal and spatial measures, and multiple technologies. To elaborate, the 100 billion neurons in the human brain, their trillions of structural and functional connections, glial structure, lesions and the electro-chemical function of the brain are captured through lenses of varying measurement types. The resulting images generate massive amounts of data so that even storage and representation of these data raise significant challenges. Furthermore, since the measurements capture the brain at multiple spatial and temporal scales, with different functional, structural, and compositional targets, the ability to synthesize this information is of fundamental importance for progress in understanding the brain and its pathologies. The term “big data” in this area encompasses this intersection of data size, complexity and modalities. Thus, efficient analysis and process of big data and the development of high-performance computing tools is critical for modern neuroscientific studies.

Despite many existing successful efforts in the analysis of large neuroimaging datasets, there remains ample room for new methods to meet these challenges. In this Frontiers research topic, we selected 14 excellent research articles that present statistical challenges and/or proposed new approaches for dealing with neuroimaging big data.

The issue boasts of a total of 60 contributors, having a wealth of experience in the area and diverse backgrounds, including: Statisticians, neuroscientists, psychologists, and computer scientists. Their insights brought statistical and computational innovations to make significant progress on the most important questions in neuroimaging. Below we provide a brief overview of all the articles in this research topic.

Functional connectomics being a fundamental area for studying neural communications represents a focus of the issue, with a wide range of topics for studying the functional connectome using resting state fMRI (R-fMRI) data. In particular, Boubela et al. have developed parallel computing algorithms and efficient implementations using apache spark and graphical processing unit (GPU) techniques for analyzing big R-fMRI data. These computational tools are quite useful for scalable analysis of very large neuroimaging datasets. Chen et al.; (Bowman et al.) have proposed a novel empirical Bayes method to normalize functional brain connectivity metrics on a posterior probability scale. This method can facilitate appropriate quantifications of existing connectivity metrics and produce reproducible scientific findings. Kalcher et al. concentrated on an interesting and important problem: Identifying venous voxels in R-fMRI data in order to increase the specificity of fMRI analyses to microvasculature in the vicinity of the neural processes triggering the blood oxygenation level dependent (BOLD) response. They solved this challenging problem by applying a graph based clustering algorithm on thresholded correlation graphs. Wang et al. studied the difference between correlation-based graphs and partial correlation based graphs in terms of estimating functional connectivity using R-fMRI data. They have developed an efficient and reliable statistical procedure based on the constrained L1-minimization Approach (CLIME) in large-scale brain networks for single subject fMRI data analysis. They also have proposed a new *Dens*-based selection method that provides a more informative and a flexible tool to allow the users to select the tuning parameter based on the desired sparsity level. For the analysis of multiple subject fMRI data, Narayan and Allen defined functional connectivity using Gaussian graphical models. They proposed a mixed-effects model that treats both subject level networks and population level covariate effects as unknown parameters. They adopted resampling based methods to improve the power for detecting the differences in multi-subject functional connectivity. Adopting an alternative modeling approach for the brain network. Li et al. have proposed to use a non-parametric independent component analysis (ICA) to separate the latent source signals from the R-fMRI data. Their novel ICA algorithm is based on density estimation and maximum likelihood, where the densities of the signals are estimated via p-spline based histogram smoothing and the mixing matrix is simultaneously estimated using an optimization algorithm. The proposed approach is very straightforward to implement and shows good performance for recovering the established brain networks. The dynamic nature of the functional connectivity was studied by Xu and Lindquist. They introduced a new data-driven algorithm to detect temporal change points in the functional connectivity and estimate a graph between region of interests (ROIs) by adopting a sparse matrix estimation approach and a hypothesis testing procedure to determine change points. This is referred as the Dynamic Connectivity Detection (DCD) algorithm which improves the recently developed Dynamic Connectivity Regression (DCR) algorithm in terms of computational efficiency and scalability for the large-scale data analysis.

In addition to the R-fMRI data analysis, the research topic also includes a new statistical approach to detecting subtle shape differences in the hemodynamic response at the group level in the fMRI studies (Chen et al.). This method estimates the shape features of hemodynamic response function using multiple basis functions and new dimension reduction methods. It is useful for improving the statistical power in detecting the brain activity signals at both the individual level and the group level.

In addition to the problems in the functional magnetic resonance imaging (fMRI) (Boubela et al.; Bowman et al.; Chen et al.; Chen et al.; Kalcher et al.; Li et al.; Narayan and Allen; Tagliazucchi et al.; Wang et al.; Xu and Lindquist), our research topic also covers a variety of other imaging modalities, such as structural magnetic resonance imaging (sMRI) (Lee et al.; Zhan et al.), diffusion tensor imaging (DTI) (Bowman et al.), magnetoencephalography (MEG) (Llinás et al.) and electorencephalograms (EEG) (Ngo et al.). Among those, Bowman et al. presented a statistical framework for analyzing neuroimaging data from multiple modalities to identify important biomarkers for Parkinson's disease (PD) risks. Their approach builds on the elastic net, performing regularization and variable selection with introducing additional criteria for parsimony and reproducibility.

Focusing on another progressive brain disease, the Alzheimer's disease (AD), Zhan et al. developed new methods to model brain structural networks from diffusion MRI and proposed a novel feature extraction and classification framework based on higher order singular value decomposition and the sparse logistic regression approach.

For the study of brain morphometry, Lee et al. developed new statistical approaches for the longitudinal regional analysis of volumes examined in normalized space (RAVENS). The method is a variant of the longitudinal functional principal component analysis (LFPCA) for high-dimensional images, which can separate registration errors from other longitudinal changes and baseline patterns, and thus address the limitations of the existing methods. Many statistical methods and computational algorithms have been developed for fMRI and MRI data analysis, limited statistical methods have been proposed to address the MEG analysis. Along this direction, we have included one article that focuses on frequency-pattern analysis of MEG data to reconstruct the brain spontaneous activities (Llinás et al.). The proposed method is among the very first to successfully characterize brain electrical activities and localize the sources in anatomical brain space in combination with MRI data. In addition to the systematic statistical approaches for analysis of big neuroimaging, we also include an exploratory data analysis approach to EEG data: The functional boxplots approach. It analyzes log periodograms of EEG time series data in the spectral domain. It identifies a functional median, summarizes variability, and detects potential outliers.

In summary, our research topic has collected a series of new statistical approaches to addressing important questions in neuroimaging big data analyses from statistically efficient, computationally scalable and scientifically meaningful perspectives. It covers a broad range of imaging modalities, including fMRI, sMRI, dMRI, DTI, EEG, and MEG. It studies a variety of mental health diseases, including Parkinson's, autism spectrum disease, Alzheimer's and multiple sclerosis.

We hope that this issue will spur discussion and open a forum for statisticians, computer scientists, neuroscientists and psychologists to further contribute the innovations in this important topic.

## Author contributions

All authors contributed equally to this content.

## Funding

BC was supported by NIH grants RO1 EB012547 and P41 EB015909 and JK was supported by NIH R01MH105561.

### Conflict of interest statement

The authors declare that the research was conducted in the absence of any commercial or financial relationships that could be construed as a potential conflict of interest.

